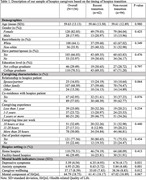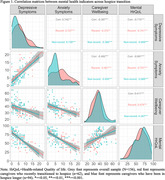# Transition to hospice: how it impacts the mental health of caregivers of persons with dementia

**DOI:** 10.1002/alz.085102

**Published:** 2025-01-09

**Authors:** Oonjee Oh, Debra Parker Oliver, Karla Washington, George Demiris

**Affiliations:** ^1^ University of Pennsylvania, Philadelphia, PA USA; ^2^ Washington University in St. Louis, St. Louis, MO USA

## Abstract

**Background:**

Within the dementia space, many caregivers lack understanding of hospice care and may not be well prepared for transition to hospice. Nonetheless, few studies have explored hospice transition specifically from the perspective of caregivers for persons with dementia and how it impacts their mental health. In this study, we aimed to examine caregivers’ mental health indicators and their correlation structure based on the timing of hospice transition.

**Method:**

We conducted a secondary data analysis using the pre‐intervention data from an ongoing randomized clinical trial of a problem‐solving therapy intervention for hospice caregivers labeled ENCODE (Empowering Caregivers of Patients with Dementia), focusing on a sample recruited between December 2021 and mid‐January 2024. Recent transition to hospice was defined as enrollment in hospice for less than 14 days at baseline. We conducted two‐sample T‐tests and χ² tests to compare the demographics, caregiving characteristics, hospice setting, and mental health indicators (i.e., depressive and anxiety symptoms, perceived change in wellbeing, the mental component of health‐related quality of life (HrQoL)) between caregivers who recently transitioned to hospice and those who had been in hospice longer. Additionally, we compared the Pearson correlation matrices across mental health indicators based on the caregivers’ hospice transition.

**Result:**

Among 156 caregivers (mean age = 59.63, female 82.05%, spouse/partner 16.03%), 62 (39.74%) caregivers recently transitioned to hospice. Compared to other hospice caregivers, we found that caregivers who recently entered hospice had significantly higher levels of depressive symptoms (p value = 0.031), higher levels of anxiety symptoms (p value = 0.001), and worse trends in overall changes in wellbeing (p value = 0.012). There were no significant differences in the mental component of HrQoL. The association patterns between each pair of mental health indicators were consistent across different timings of hospice transition.

**Conclusion:**

In the context of dementia care, our results highlight that caregivers who just entered hospice are undergoing a challenging transition that often finds them in a mentally vulnerable position. To develop and implement effective strategies for caregivers of persons with dementia, we need to understand the needs and vulnerabilities of caregivers during hospice transition and identify the best timing for the delivery of supportive tools.